# The invisible lifeline: Intricacies of the global plasma supply

**DOI:** 10.1002/ajh.27499

**Published:** 2024-10-06

**Authors:** Jan Hartmann, Sean R. Stowell, Harvey G. Klein

**Affiliations:** 1 Global Medical Office, Haemonetics Corporation, Boston, Massachusetts, USA; 2 Department of Pathology, Joint Program in Transfusion Medicine, Brigham and Women’s Hospital, Harvard Medical School, Boston, Massachusetts, USA; 3 Qualtex Laboratories, Norcross, Georgia, USA; 4 Department of Transfusion Medicine, Clinical Center, National Institutes of Health, Bethesda, Maryland, USA

## 1 | INTRODUCTION

Like whole blood, plasma is collected from healthy human donors. However, while much is known and has been written about whole blood donations and the worrisome trends that increasingly challenge its safety and global supply,^1–4^ the complexities of plasma collection and its supply chain—from the donor’s arm, through the manufacturing process, to the patient’s therapy—have, with few exceptions,^5–8^ received far less attention. Yet the biological medicines prepared from plasma represent a critical lifeline for an increasing number of patients worldwide.

## 2 | SCARCE YET ESSENTIAL RESOURCE

Human plasma forms the indispensable source material for the drugs that are known as plasma-derived medicinal products (PDMPs). Plasma can be obtained through two processes. “Recovered plasma” is the by-product of separation of whole blood into its component parts. Recovered plasma constitutes a minor fraction of the plasma used to manufacture PDMPs. Most of the plasma intended for fractionation into PDMPs is collected by plasmapheresis and is known as “source plasma.” The frequency and volume of source plasma donations are strictly regulated by national regulatory authorities, for example, by the FDA in the United States, the Paul-Ehrlich Institute in Germany, and the Therapeutic Goods Administration in Australia. Most European blood establishments follow the guidelines of the European Directorate for the Quality of Medicines & HealthCare (EDQM).

More than 30 proteins are currently prepared by fractionating plasma. Many of these PDMPs are deemed sufficiently important to a nation’s healthcare system that they have been included in the WHO’s Model List of Essential Medicines for Adults and Children. The list of therapies derived from human plasma is long, as is the list of conditions treated by them.^8^ The range of indications includes hematological conditions, such as hemophilia, von Willebrand disease, ITP, anti-thrombin III deficiency, HIT, VITT, secondary immunodeficiency in CLL patients and patients undergoing cancer chemotherapy, neurologic, renal, and autoimmune disorders. The list and number of patients continue to grow as does worldwide demand.^9–11^

Many donors and donations are needed to fulfill the current plasma demand. It is estimated that treating one hemophilia A patient for a year requires plasma from approximately 1200 donations (US Department of Health and Human Services, “Why Giving Plasma is So Critical”; retrieved on 9/20/2024 from: https://www.hhs.gov/givingequalsliving/giveplasma/why-give). The World Federation of Hemophilia estimates that 400 000 people are living with hemophilia and only 25% receive adequate treatment.^12^ Also, the need for immunoglobulin is growing.^13–15^ This trend is driven by demographic changes, such as longer life expectancy and increased average weight, by higher diagnostic and treatment rates, and increasing demands in low- and middle-income countries (LMICs). Furthermore, expanded uses are expected, including for immunodeficiencies and for off-label uses in indications where the safety and efficacy have not yet been formally established.

As with other limited resources, access to PDMPs, especially immunoglobulin, can be restricted even in high-income countries, requiring prioritization or trade-offs between patient groups. Outright shortages, for example, of immunoglobulin preparations, have occurred numerous times over the last few years. These shortages typically develop through supply disruptions, are mostly unpredictable, and are often recognized too late to make provision for adequate patient care.^16,17^

While there are regular efforts to raise awareness of whole blood shortages and campaigns to recruit and retain whole blood donors, plasma shortages often go unnoticed by the general public and appeals to increase plasma donations are rare. The recruitment of convalescent plasma donors during the recent COVID-19 pandemic has been a short-lived exception that brought heightened visibility to plasma as a therapeutic entity.^18,19^ However, as the pandemic waned, so did the public’s concern for the need for plasma. Whereas collections of whole blood donations rebounded quickly, that was not the case for plasma donations.^7^ A broader understanding of the challenges of the global plasma supply chain may help raise awareness and facilitate improvements.

## 3 | RISKS OF A CONCENTRATED SOURCE PLASMA DONOR POOL

The global plasma supply depends on the availability of donors. Before the COVID-19 pandemic, over 70% of source plasma was collected in the United States (PPTA, The Source, Fall 2019; retrieved on 9/20/2024 from: https://drive.google.com/file/d/1p2fHo58muiQlpPfVHhBDWLnGEWtIskUy/view). In fact, blood and blood components make up approximately 2.5% of US exports, according to the US Census Bureau, with much of that being plasma. And even within the United States, the plasma donor pool is concentrated among a very small percentage of paid donors. There are over 1000 commercial donation centers across the United States, with the highest density along the southern border with Mexico and around college campuses.

The concentration of the plasma donor pool by geography and socioeconomic profile poses a significant risk to the overall plasma supply. For example, during the COVID-19 pandemic, the number of plasma donations in the United States dropped significantly.^7^ Even regional events impacting the United States, such as the recent closure of border crossings with Mexico, had a measurable effect on plasma collections, impacting the supply chain globally. Many countries, particularly in Europe, are aware of their strategic dependency on the United States and are striving for independence. However, other than Germany, Austria, Hungary, Egypt, and the Czech Republic, no other country has reached self-sufficiency. The World Health Assembly resolution WHA63.12 directs the WHO to promote self-sufficiency in the supply of safe blood components using voluntary blood donations whenever possible and advocates “security of supply” as an important goal to prevent blood shortages and to meet national transfusion requirements. However, this goal is more easily decreed than achieved.^20^

While blood safety has improved tremendously since the HIV/AIDS crisis in the 1980s, blood-borne pathogens remain a credible threat. The potential transmission of a novel or epidemic pathogen via blood or plasma donations could halt plasma collections in the United States as it did in the United Kingdom in 1986. The detection of a previously unrecognized transfusion-transmissible pathogen, a prion causing variant Creutzfeldt-Jakob (“mad cow”) disease, resulted in termination of all plasma collections in the United Kingdom and Ireland and necessitated plasma importation from the United States to meet UK patient needs. The COVID-19 pandemic has demonstrated that even without blood-borne transmission, a pathogen can severely disrupt the donation infrastructure.

In addition to infectious threats, there are IT-related risks including cybersecurity threats. Cyber-attacks have the potential to significantly disrupt the operations of one or more collection organizations, which could lead to a noticeable decrease in collection volumes.

Finally, the number of commercial collectors and fractionators has been reduced because of mergers and acquisitions and the size of the resulting companies has grown significantly. Commercial plasma collection centers in the United States and Europe have been incorporated into fractionation companies to improve efficiency by vertical integration. Such consolidation further raises concerns that a manufacturing interruption at a single company or one of its suppliers could have a devastating effect on the global PDMP supply.

## 4 | RECRUITMENT CHALLENGES

While volunteer whole blood donors often return over extended periods, the average source plasma donor in the United States remains active for under 1 year. This means that constant recruitment efforts are needed to attract donors. Plasmapheresis requires a much longer time commitment than does whole blood donation. Source plasma donors historically have been younger, more often male, and of a lower socioeconomic stratum than that of whole blood donors. In the United States, where remuneration is allowed, financial incentives have greatly aided the regular recruitment of new and continuing donors. Other countries, except for Egypt, the aforementioned four European countries, and parts of Canada, do not allow remuneration and continue to experience recruitment issues.

To better assure safety, plasma from newly recruited plasmapheresis donors can only be used after a second donation has cleared their infectious disease status. For a variety of reasons, this is not the case for whole blood donors. If the plasmapheresis donor does not return, the donation cannot be used and must be discarded. These so-called “orphan donations” are believed to account for approximately 5% of collections in the United States.

## 5 | GLOBAL DISPARITIES

The infrastructure required to collect and process plasma is highly capital-intensive, making it cost-prohibitive for many LMICs. In the United States, a plasma collection center typically requires millions of dollars to build and operate, while facilities for plasma fractionation can cost over $1 billion. Most LMICs lack nationally coordinated blood systems and typically depend upon hospital-based collections. Screening for markers of infectious disease is often inadequate. The absence of inspection and licensing of the blood establishments by national regulatory authorities results in noncompliance with Good Manufacturing Practice and plasma quality that does not meet international fractionation standards. WHO has encouraged LMICs to make better use of their recovered plasma. Although the several million units of recovered plasma should not be wasted, recovered plasma alone will not suffice to meet the fractionation needs of LMICs (WHO Guidance on increasing supplies of plasma-derived medicinal products in low- and middle-income countries through fractionation of domestic plasma, 2021; retrieved on 9/20/2024 from: https://apps.who.int/iris/handle/10665/3401).

As a result, there are only around a dozen large-scale fractionation sites across the globe, most of them in the United States and Europe. This concentration, further aggravated by corporate consolidation, highlights the risk of significant disruptions if one of those sites is compromised, and raises logistical and ecological challenges related to transporting tens of millions of liters of frozen collected plasma across long distances from collection sites in the United States to fractionation sites overseas.

The lack of large-scale plasma collection and fractionation infrastructure in LMICs, relative to the large populations, effectively excludes a significant portion of the global population from donating plasma.^21^ Likewise, the benefits of PDMPs are not shared equitably across the globe. Many LMICs lack basic access to PDMPs. Recently, the Egyptian government has made a concerted effort to build collection infrastructure and has achieved a level of self-sufficiency. For now, the country remains dependent on fractionation capacity but is in a much better position to provide its citizens with access to PDMPs.

## 6 | OPPORTUNITIES TO EXPAND DONOR POOL

The most effective way to secure and increase the global plasma supply is to grow the donor pool through geographic expansion and effective donor recruitment and retention. The concentration of global donations in the United States is a weakness in the system and a strategic risk to countries outside the United States. While the European Commission has acknowledged this risk, Europe is struggling to effectively address it. Initiatives for self-sufficiency should be prioritized, and successful strategies from countries that have achieved self-sufficiency should be adopted. Following the example of Egypt, more LMICs could be encouraged to participate in the global donor pool. More innovative collection and lower cost fractionation models allowing decentralized processing are needed.^21^ WHO has recently issued guidance on increasing supplies of plasma-derived medicinal products in LMICs through fractionation of domestic plasma (retrieved on 9/20/2024 from: https://www.who.int/publications/i/item/9789240021815). In the United Kingdom, plasma donations prior to 2021 could not be used for fractionation as a precaution against possible risks stemming from variant Creutzfeldt-Jakob disease. After the ban was lifted in 2021, the NHS resumed collections, resulting in beneficial effects on the plasma and PDMP supply in Europe.^22^

Beyond geographic expansion, investment in donor recruitment and retention is crucial. Using the example of the United States, the donor pool is very limited, and different strategies and methods may be required to approach and convince new donors, particularly those outside of the historic demographics. Retention efforts are equally important. Focus should be placed on donor comfort and experience to encourage repeat donations.^23,24^

Importantly, remuneration appears to be a critical driver for large-scale donor turnout. This approach must be weighed against potential challenges. Arguments against remuneration include ethical concerns like the risk of donor exploitation, opposition to the sale of human tissues, concerns about donor health, worries about competition with whole blood donation efforts, and unease about the safety and quality of the source plasma pool if donors are financially incentivized to donate. While there are valid counterarguments and studies addressing these concerns, the issue of remuneration for source plasma donors needs to be taken seriously and deserves a contemporary, open, and fact-based debate. Ultimately, these challenges must be weighed on an individual and societal level against the benefits of donations and the potential harm to patients if PDMPs are not available.

## 7 | IMPORTANCE OF EVERY DONATION

As donations are so valuable and the recruitment and retention of donors are so challenging, maximum benefit should be derived from each donation opportunity. New, more personalized approaches to source plasma collection have been proposed^25,26^ and recently studied in a large-scale randomized clinical trial.^27^ Under such personalized schemes, donors are assessed individually before each donation, and the target volume for their collection is determined by factors such as weight, height, and hematocrit. Based on that, donors with less expected plasma volumes donate less, and donors with higher plasma volumes can donate more. In a recent randomized controlled trial, this approach yielded approximately 8% more plasma on average per donation. Donor safety data demonstrated that the personalized approach was noninferior to the control plasmapheresis method.^27^ The safety profile of this method has been further supported by the analysis of a recent real-world data set of close to five million source plasma collections.^28^

In view of the worldwide plasma shortage, it is regrettable that “orphan donations” must be discarded, resulting in the waste of a valuable resource. Given the current highly sensitive donor screening and testing and the safety measures developed for the plasma fractionation process that can effectively eliminate pathogens, it seems timely to reassess the benefit–risk profile of this strategy. Regulators in the United States and abroad should prioritize this important topic.

## 8 | MORE TRANSPARENCY

Lastly, a significant cause of the anxiety regarding medication availability, for both patients and caregivers, stems from a lack of transparency about the supply. Given the long lead time to produce some of these PDMPs, approximately 6–12 months from plasma collection to distribution, it would be helpful to develop leading indicators that could signal looming drug shortages. This should include a current understanding of available safety stocks that can buffer up to a few months of source plasma supply disruption. Due to competitive sensitivities between commercial plasma collection organizations and pharmaceutical companies, this could perhaps be best managed through an independent third party that only provides aggregated information.

Similarly, reliable demand forecasting on the clinical use in various indications and the resulting need should be encouraged and funded. This is particularly important for treatments that depend on immunoglobulin, currently the most needed therapeutic extracted from plasma.

## 9 | CONCLUSION

The global plasma supply chain is vital, yet complex and fragile. As the number and need for PDMPs grow, this invisible lifeline requires our attention and action. To ensure PDMP availability, we must expand the donor pool, address remuneration challenges, reduce the waste of orphan donations, investigate novel methods of processing surplus recovered plasma from LMICs, and enhance transparency as well as supply and demand forecasting. By fostering international collaboration and adopting successful models, we can build a more resilient and equitable plasma supply system that works toward making PDMPs accessible to all in need.

## Data Availability

Data sharing is not applicable to this article as no new data were created or analyzed in this study.
